# Extracellular Vesicles and Their Convergence with Viral Pathways

**DOI:** 10.1155/2012/767694

**Published:** 2012-07-25

**Authors:** Thomas Wurdinger, NaTosha N. Gatson, Leonora Balaj, Balveen Kaur, Xandra O. Breakefield, D. Michiel Pegtel

**Affiliations:** ^1^Departments of Neurology and Radiology, Massachusetts General Hospital and Neuroscience Program, Harvard Medical School, Boston, MA 02129, USA; ^2^Neuro-oncology Research Group, Department of Neurosurgery, VU University Medical Center, De Boelelaan 1117, 1081 HV Amsterdam, The Netherlands; ^3^Dardinger Laboratory for Neuro-oncology and Neurosciences, The Ohio State University, Columbus, OH 43210, USA; ^4^Department of Pathology, Cancer Center Amsterdam, VU University Medical Center, 1081 HV Amsterdam, The Netherlands

## Abstract

Extracellular vesicles (microvesicles), such as exosomes and shed microvesicles, contain a variety of molecules including proteins, lipids, and nucleic acids. Microvesicles appear mostly to originate from multivesicular bodies or to bud from the plasma membrane. Here, we review the convergence of microvesicle biogenesis and aspects of viral assembly and release pathways. Herpesviruses and retroviruses, amongst others, recruit several elements from the microvesicle biogenesis pathways for functional virus release. In addition, noninfectious pleiotropic virus-like vesicles can be released, containing viral and cellular components. We highlight the heterogeneity of microvesicle function during viral infection, addressing microvesicles that can either block or enhance infection, or cause immune dysregulation through bystander action in the immune system. Finally, endogenous retrovirus and retrotransposon elements deposited in our genomes millions of years ago can be released from cells within microvesicles, suggestive of a viral origin of the microvesicle system or perhaps of an evolutionary conserved system of virus-vesicle codependence. More research is needed to further elucidate the complex function of the various microvesicles produced during viral infection, possibly revealing new therapeutic intervention strategies.

## 1. An Introduction to Extracellular Vesicles

**Figure 1:**
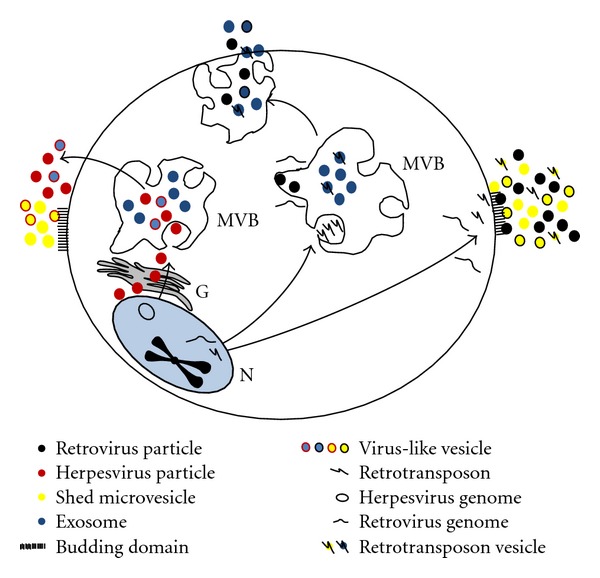
Convergence of microvesicle and virus biogenesis. Viruses share effectors of microvesicle production for their assembly and release. Exosomes produced in the MVB and shed microvesicles budding of the plasma membrane are indicated by blue and yellow dots, respectively. Extrachromosomal herpesvirus genomes are indicated by circles, retroviral genomes by sea-gull wings, and retrotransposons by the Y drawing. Herpesviruses, retroviruses, and retrotransposons sharing exosome or shed microvesicle pathways are indicated by red, black, or Y-containing dots, respectively. Chimeric virus-like vesicles are exosomes or shed microvesicles containing viral or retrotransposon elements and are indicated in dual color. N: nucleus, G: Golgi apparatus, MVB: multivesicular body.

A wide variety of vesicles are actively released from living cells into the extracellular space with their contents reflecting the cellular composition and physiologic state (for review see [1–3]). Over the years, the different types of extracellular vesicles have been given a variety of names, including exosomes, shed microvesicles, ectosomes, microparticles, virosomes, virus-like particles, and oncosomes. The distinguishing features of each of the vesicle subtypes and the correct nomenclature are currently under intense study. Here, we will refer to them under the general term, microvesicles. Microvesicles carry RNA [mRNA, microRNA (miRNA), and noncoding sequences], cDNA and genomic sequences, and a large component of proteins and lipids (see reviews above, as well as [4, 5]). Upon release these microvesicles can move within the extracellular space and are either taken up by neighboring cells or degraded. They can also enter adjoining bodily fluids, such as the systemic circulation and travel to distant sites. In fact, they have been found in abundance in blood (serum and plasma), urine, breast milk, sweat, saliva, ascites fluid, and cerebral spinal fluid (CSF) [3–7]. At least two distinct release mechanisms for microvesicles have been described for two subtypes: (1) exosomes—derived from the multivesicular body (MVB) and (2) shed microvesicles—derived from the plasma membrane. Interestingly, both mechanisms have considerable overlap with virus release and biogenesis (summarized in Figure 1 and further discussed below). 

Exosomes range from 30 to 100 nm in diameter and are generated by inward budding of the lumen of internal vesicular compartments derived from endosomes [8]. As vesicles accumulate within these endosome-derived compartments, they are referred to collectively as MVBs. These MVBs can either be targeted for degradation through the lysosomal pathway, or they can fuse with the plasma membrane releasing their interior vesicles into the extracellular space. The exact mechanism and kinetics of these fusion and release events are not fully elucidated and may vary among different cell types [9]. For example, depletion of Hrs (an ESCRT-0 component) led to a decrease in exosome secretion in dendritic cells that were stimulated to release with ovalbumin and a calcium ionophore [10]. Oligodendrocytes on the other hand seem to secrete exosomes by a mechanism that is ESCRT independent and ceramide dependent [11]. Exosome release by HeLa cells has been found to involve Rab27a/b [12], and p53 is reported to play a role in exosome release in a nonsmall cell lung cancer cell line [13]. Rab11 has also been shown to be involved in the release of exosomes from MVBs by acting in the tethering/docking of MVBs to the plasma membrane to promote homotypic fusion, in the presence of calcium [14]. In addition, TBC1D10A-C, a Rab35 inhibitor, led to intracellular accumulation of endosomal vesicles and impaired exosome secretion [15]. 

Shed microvesicles are released by outward budding directly from the plasma membrane and tend to be larger (>100 nm in diameter) and more heterogeneous in size [16, 17]. Moreover, this release process is likely controlled by localized cytoskeleton dynamics, with small cytoplasmic membrane-covered protrusions detaching and being released into the extracellular space [18] by an activated GTPase, ARF6 [19]. Interestingly, recent observations indicate that virus-independent budding from the plasma membrane can be mediated by endosome to plasma membrane relocation of TSG101, a prominent member of the ESCRT-I complex, frequently noted as an exosome marker [20]. This type of budding is topologically identical to both the inward budding of the limiting membrane of MVBs and viral assembly at the plasma membrane, in that the outer surface of the plasma membrane is on the outer surface of the microvesicle. In fact, certain tumor cells shed retroviral-like vesicles, which can be abundant because of increased transcription of endogenous retroviral sequences [17, 21], resulting from overall hypomethylation of the genome [22]. In general it seems that the clear distinctions between viruses and microvesicles based on composition and function are fading although they can be separated from vesicles released during the later stages of programmed cell death since these latter vesicles, referred to as apoptotic blebs [2], are even larger in size [23].

The role of microvesicles in intercellular communication is currently receiving much attention. Upon release from the donor cell, the microvesicles can either be taken up by neighboring cells or travel through bodily fluids for cargo delivery into recipient cells at distant sites. Although many details are missing, cellular uptake of some microvesicles appears to depend, at least in part, on specific ligand-receptor recognition [24], and can be mediated by direct fusion of the microvesicles with the plasma membrane or by endocytotic uptake of the microvesicles. For example, Quah et al. [25] have shown that bystander naïve B cells are rapidly activated by acquiring the antigen from activated B cells through microvesicle-mediated membrane transfer. In a similar way CD41 is transferred from platelets to endothelial and tumor cells, resulting in increased proadhesive properties of the recipient cells [26, 27]. Microvesicles also shuttle mRNA between cells and influence the physiological state of the recipient cell, as well as the cellular response to external stress stimuli [28]. In addition, miRNAs are transferred by exosomes [6, 29, 30]. For instance, miR-146a was shown to be transferred into recipient prostate cancer cells leading to the inhibition of their proliferation [31], and recently miRNAs which can modulate the immune response were detected in exosomes in breast milk [32]. Furthermore, retrotransposon sequences are particularly enriched in tumor microvesicles, and tumor-derived human endogenous retroviral (HERV) sequences can be transferred to normal human umbilical vein endothelial cells (HUVECs) via microvesicles resulting in a prolonged increase in HERV-K mRNA levels [17]. This suggests that tumor cells transfer these mobile genetic elements via microvesicles to neighboring normal cells thereby modulating their genotype and phenotype. 

## 2. Viruses and Microvesicles

**Figure 2:**
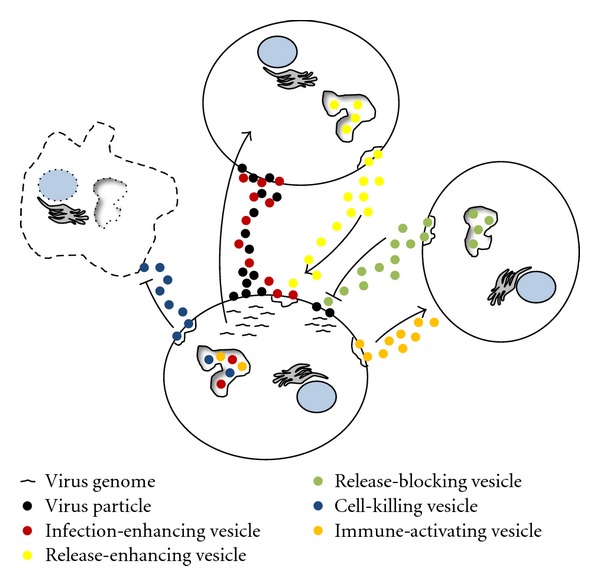
Heterogeneity of microvesicle function during virus infection. Microvesicles with diverse effects on virus spread are indicated by different colors. Microvesicles from infected cells can affect noninfected cells, enhancing infection, or killing incoming immune cells, or act to activate immune cells to viral antigens. Microvesicles from noninfected cells can either enhance or block virus release from the infected cell and modulate the immune response. Cell with dashed lines is undergoing cell death.

Microvesicular shedding of cellular membrane components and the release of internal endosomal-derived exosomes are important for cellular communication and modulation of immune responses [9, 54–57] (Table 1). While release of microvesicles has been extensively investigated, recently the challenge has been to uncover the specific mechanisms that guide protein sorting and complexing into shed microvesicles and exosomes in various cell types. Cells have been reported to secrete highly specified microvesicles after infectious exposure or under various cell activation conditions [5, 54, 56, 58]. Through the packaging and transfer of functional proteins, mRNA/miRNA, and other cytosolic components, microvesicles have been found to be beneficial either to the host cell or to the infectious agent [37, 43]. Virus-infected cells proved useful in early studies to elucidate the role of microvesicular shedding in intercellular communication [55, 56]. Amongst the most extensively studied viruses with respect to microvesicles are herpes simplex virus (HSV), human immunodeficiency virus (HIV), and the tumorigenic herpes virus, Epstein-Barr virus (EBV). Each virus possesses unique properties that afford protection from immune attack. Here, we outline the important immune modulatory steps involved in virus-induced microvesicle sorting and release in these and other related viruses. Preservation of the virus depends on microvesicle release of infected cells. Microvesicles released by infected cells contain specific components of the cell and the virus, many of which facilitate the ability of virions to persist in a hostile antiviral immune environment [44, 55, 56, 58]. Depending on the virus type, and, in some cases, the stage in the viral cycle, intercellular processes are well orchestrated to produce specific cellular and immune outcomes [56]: (1) evading the host immune system, (2) invasion, (3) replication, and (4) persistence (summarized in part in Figure 2 and further discussed below).

### 2.1. Evading the Host Immune System

During primary viral infection, humoral and cell-mediated host immune responses such as production of neutralizing antibodies and cytotoxic T-cell attack on infected cells are employed to contribute to viral destruction. Early evasion strategies adopted by viruses interfere with complete elimination of the virus, allowing it to persist. During HSV-1 infection the release of microvesicles, formerly known as L-particles containing viral tegument proteins and glycoproteins, can prime surrounding cells for productive infection and reduce immune rejection [48–50]. Such virus-like vesicles lack both the viral capsid and DNA and are thereby incapable of producing a replication-infective cycle in the cells on their own [49–51]. However, some of the viral tegument proteins contained within them are immediate early transcription factors that can produce rapid transcriptional activation of later arriving intact virions [48, 52]. Another evasion strategy observed for HSV-1 is targeting of the MHCII molecule processing pathway by viral envelope glycoprotein B (gB) [37]. Antigen-presenting cells (APCs) routinely sort the MHCII surface receptor HLA-DR to MHCII compartments for processing. The primary role of this pathway is to present peptide antigens to the immune system in order to elicit or suppress T-(helper) cell responses that stimulate B-cell production of antigen-specific antibodies [37]. HSV-1 gB couples with HLA-DR, causing sorting through the exosome pathway as opposed to presentation on the cell surface. Complexing of gB-DR effectively hijacks the cellular antigen presenting machinery, preventing further peptide loading and, in addition, increasing microvesicle production [37, 53]. This final step releases additional gB-DR complexes into the host immune microenvironment, promoting resistance of viruses to immune attack, and in some cases producing bystander T-cell tolergenicity or anergy [37, 53]. In the case of HIV, microvesicle packaging and spread of the virus-encoded Nef protein impairs proper endocytosis of the immature MHCII/invariant chain, antibody class switching, and lysosomal degradation of viral peptides allowing HIV virus to evade immune recognition [37, 38]. EBV, human cytomegalovirus (CMV) and hepatitis C virus (HCV) have also found means to evade immune responses by exploiting microvesicles, as discussed below.

### 2.2. Invasion and Replication within the Host Cell

Exosomes and shed microvesicles can both incorporate elements from the cell, as well as from the intruding virion [54]. Upon circulation of these microvesicles, they encounter and enter susceptible cells and can sensitize them to viral infection thus increasing systemic spread of the virus to naïve cells. In the case of the human CMV, microvesicles released by infected cells present the C-type lectin family molecule expressed on dendritic cells—used in capture and internalization of pathogens—in complex with the CMV glycoprotein B. This complex can be subsequently distributed to other cells by microvesicles, thereby increasing the susceptibility of these cells to CMV [59]. A similar mechanism is found in the case of HCV. In HCV-positive patients, the cellular membrane protein CD81 associates with one of the HCV envelope glycoproteins, E2. Extracellular release of the E2-CD81 complexes within microvesicles allows for increased virus-fusing ability and infectivity of previously naïve cells [60]. Microvesicles bearing the E2-CD81 complex and containing HCV RNA are of notable importance as they have been reported to be infectious even in the presence of neutralizing antibodies [60]. Interestingly, HCV has been shown to release three phenotypically distinct types of microvesicles having variable infectivity from high to low [60]. However, differential release of these microvesicles during HCV pathogenesis remains to be elucidated.

### 2.3. Microvesicles Contribute to Host Immunity against Viral Infection

Conversely, microvesicular release can contribute to viral attack by the host immune system. For example, in early invasion steps of CMV, CMV antigens are transferred from infected epithelial cells (ECs) via EC-derived microvesicles to APCs [43]. These APCs are not detected as infected cells but are rendered more susceptible to infection with subsequent encounters with the virus [43]. While this is a primary infectious viral invasion and replication strategy, inadvertently transferred APCs bearing CMV antigens in transplanted organs serve as markers to the host immune systems to target nonself tissue. Harboring of these susceptible APCs by the immune-compromised host and continued microvesicular shedding increases T-cell surveillance and influx into the grafted tissues, thereby exacerbating allograft rejection [43]. Microvesicles can also promote the innate immune response to viruses, for example, as observed for HIV whereby transfer of a particular antiviral cytidine deaminase via exosomes inhibits HIV replication [61]. In addition, virus-like vesicles can be used as a vaccination strategy, and recently chimeric virus-like vesicles were engineered using a mixture of coronavirus and influenza proteins functioning as a potential severe acute respiratory syndrome (SARS) virus vaccine [62].

### 2.4. Further Applications

Viruses can use various microvesicle transport mechanisms as a survival strategy, while in other cases the host immune system can utilize microvesicles for cell signaling and host protection. Microvesicles can directly activate or suppress cellular responses, induce or facilitate infection, and transfer material to improve or hinder host immune recognition [9]. These same strategies can be exploited in the development of virus-based therapies. Oncolytic viruses armed with therapeutic genes are currently being evaluated for safety and efficacy for cancer therapy [63–65]. It would be of interest to determine whether microvesicles can alter the efficacy of oncolytic viruses, and other types of viral gene delivery vectors. Recent work shows that microvesicles can be loaded with adenoassociated viral (AAVs) vectors for more efficient gene delivery [66], opening a new window into the microvesicle therapeutics field.

## 3. EBV and Microvesicles

Several human pathogenic viruses are known for their ability to lie dormant in the host immune system, of which HSV and EBV are perhaps the best known examples. In the case of HSV this is due to the ability of the virus to enter a latent state in the nucleus of sensory neurons during which it expresses no viral antigens and does not disturb the physiology of the neurons. In latency a single transcript is generated which encodes a precursor for four distinct HSV, miRNAs which act to suppress virus replication [67]. For human herpesvirus 4 (HHV4), better known as EBV, this is largely due to incomplete eradication of the virus after early primary infection.

Gamma herpesviruses, including EBV, have developed a variety of strategies to exploit host-cell regulatory pathways that lead to a permanent infection of their host. When these pathways are deregulated, what is usually an undamaging herpes infection can predispose to disease-including encephalitis, autoimmunity, and cancer [68]. It was recently demonstrated that EBV exploits the endosomal-exosomal pathway by balancing intracellular signaling in infected B cells [69] and controlling epigenetic changes in uninfected neighboring cells via microvesicles [30]. Enveloped viruses of the herpes virus family, such as human CMV (HCMV/HHV5) and EBV, depend on the interaction with cellular endosomal membrane systems for replication [70]. Interestingly, mature HHV-6 virions are released together with internal vesicles through MVBs by the cellular endosomal-exosomal pathway [71]. Thus, many herpesviruses generally seem to exploit endosomal pathways and microvesicles for virus production, release, and immune evasion. However, the finding that viruses such as EBV modulate host-cellular pathways that are not directly involved in virus production needs further investigation.

Being the first human tumor virus identified, EBV is in many aspects an extraordinarily benign pathogen and is best known as the causative agent of “kissing disease” or infectious mononucleosis. It is estimated that over 90% of the world population is persistently infected with EBV. The EBV life cycle begins by exchange through saliva and EBV virions that seem to preferentially infect naïve resting B cells in secondary lymphoid organs, such as the tonsils. Occasionally isolated epithelial cells also become infected and presumably sustain lytic replication [72], which is required for viral shedding into the saliva for transmission to new hosts [73]. To reach its near universal prevalence without harming the host, EBV and related persistent herpesviruses have evolved complex strategies encouraging immune recognition in proliferative (potentially oncogenic) stages of its life cycle, while elegantly avoiding the immune recognition at other stages by “going into hiding” [74]. Upon initial infection at the mantle zone of germinal centers (GCs), the newly infected naïve B cells undergo multiple differentiation stages and tight interactions with surrounding stroma and T cells [75]. Interestingly, EBV facilitates these essential interactions for the maturation of B cells, for instance, by upregulation of crucial GC reaction-associated proteins, such as GP183 [76]. This integral part of the EBV life cycle (i.e., mimicking a GC-type reaction) requires tight growth regulation in a specific EBV latency gene expression program (Latency III) and promotes rapid growth and proliferation of these infected cells through NF*κ*B activation. This strategy in expanding the infected pool of B cells without the need for lytic replication may be advantageous under normal conditions but raises the chances of turning-on malignant growth if the viral latency programs are not properly controlled. Indeed, if these cells do not progress further into memory cells by shutting down this growth program, they can remain in the proliferative phase and give rise to EBV-positive lymphomas which can kill the host, thus, restricting further viral propagation and spread [77]. In addition, EBV infection at this stage may also predispose to autoimmunity as inappropriate survival signals may interfere with negative selection of self-reactive B cells. Of note, immune-suppressed individuals are at increased risk for developing EBV-driven lymphomas, reflecting the importance of a lifelong potent anti-EBV T-cell response [78]. The ability of EBV to persist despite such vigorous T-cell responses indicates that EBV can escape from the adaptive immune system and may do so in part by exploiting the endosomal-exosomal pathway through the secretion of T-cell inhibitory exosomes [44–46]. When secreted by EBV-positive tumors, these exosomes carry immune-evasive proteins including the viral protein LMP1 [79] and high amounts of galectin 9 that cause massive apoptosis of EBV-specific CD4+ T-cells via specific interaction with T-cell immunoglobulin mucin-3 (Tim-3), which can negatively regulate Th1 T cell and macrophage activation. The inhibition of anti-EBV immune responses is believed to promote the progression of EBV-positive malignancies, such as Hodgkin's disease (HD) [46] and nasopharyngeal carcinoma (NPC) [80].

Vallhov et al. [81] studied the interaction between exosomes secreted by EBV-driven lymphoblastoid cell lines (LCLs) and peripheral blood B cells proliferating *in vitro*. LCLs are 95% latent, but a small proportion of cells is in a lytic stage. Exosome-cell interactions could be inhibited by specific antibodies against gp350 the major envelope protein of EBV or CD21 on B cells, indicating an interaction between CD21 on B cells and the gp350 on exosomes [81]. These specific exosome-cell interactions may be exploited for exosome-based anticancer therapies, for example, in delivering the CD154 protein to leukemic B blast cells rendering them immunogenic to T cells [82]. In addition to proteins, it is now clear that microvesicles from many cell types carry and transport functional RNA molecules. EBV was the first virus discovered to encode its own small regulatory miRNAs [83]. EBV encodes a staggering 44 viral miRNA species, derived from two major gene clusters on the viral genome, which have an important role in EBV persistence [84]. Next generation sequencing indicates that these EBV-encoded miRNAs make up a large fraction (20–25%) of the total cellular miRNA in EBV-infected cells, encompassing 300+ different miRNA species [85]. Similar results were found in the miRNA profile of exosomes from EBV-driven LCL cells (Pegtel et al., unpublished results). This is consistent with the idea that viral miRNAs manipulate gene regulation in host cellular pathways and also exploit the exosomal miRNA communication pathways.

Indeed, the discovery of EBV-encoded regulatory miRNAs (EBV-miRNAs) residing within the lumen of exosomes indicated a novel mechanism by which exosomes can exert inhibitory effects, namely, by translational repression of target genes in noninfected recipient cells via exosomal EBV miRNAs [30]. Earlier studies in mice had suggested that intact exosomes from EBV-infected cells had strong physiological effects *in vivo*, consistent with the idea that the luminal content of exosomes is biologically significant, apart from the proteins and lipids that make up their surface [86]. Subsequent studies demonstrated that EBV-infected cancer ECs also secrete EBV-miRNAs, presumably within exosomes [87]. Due to the lack of an accurate *in vivo* model for human EBV infection it is difficult to investigate the mechanism controlling release of EBV-miRNAs through exosomes and to determine whether this contributes to viral persistence in healthy infected individuals. However, EBV-encoded miRNAs are transported from infected B cells to noninfected (EBV-DNA negative) T cells and monocytes, supporting the idea of horizontal miRNA transfer in humans. Thus, viral miRNAs in exosomes may contribute to sustain persistent virus infection by delivery of such miRNAs into noninfected responding T cells leading to their inactivation (anergy) [45] or destruction [44]. This is consistent with recent data suggesting that exosomes efficiently transport miRNAs through the immunological synapse during interactions of T cells with APCs [47], similar to what is known concerning antigen exchange [88]. Studies are underway to establish whether EBV exploits these specialized intercellular contacts for efficient posttranscriptional control in neighboring responding immune cells as a possible mechanism for immune escape.

## 4. HIV and Microvesicles

HIV [56, 89–91] has been a discussion topic in the microvesicle field for many years. Not only has it been hypothesized that HIV itself may have microvesicle features, but microvesicles also have been described to have immune modulatory functions on HIV-infected cells and to expand the infectivity of HIV.

In 2003 Gould et al. [92] postulated that HIV—an enveloped retrovirus—hijacks the microvesicle system to benefit its own assembly and subsequent exit. Interestingly, inhibitors were identified that blocked the budding of both shed microvesicles and HIV particles [93]. In addition, peptides were identified that prevented interactions of HIV Nef protein—a key protein in the HIV life cycle—with mortalin, a cellular heat shock protein, and resulted in inhibition of the release of HIV and Nef-containing microvesicles [94]. Careful analysis, however, has indicated that although HIV exploits certain proteins that also play a role in exosome formation via the MVB [95], HIV assembly does not necessarily use the same logistics system as do exosomes. Importantly, it has been established that HIV budding occurs mostly at the plasma membrane and not from within the MVB [96–99]. Interestingly, HIV recruits members of the MVB ESCRT complex for proper HIV budding from the plasma membrane [98–102]. While in CD4+ T cells HIV release appears to be independent of exosomes [103], in monocyte-derived macrophages HIV can bud into endosomes [102, 104]. However, several studies highlight that HIV-1 budding also in macrophages occurs primary at the plasma membrane [105–107]. Thus, the controversy about the site of productive virus assembly in macrophages mostly favors the plasma membrane. HIV release in dendritic cells may be triggered by signals similar to those for exosome release [102, 108, 109], and secretion of HIV from endocytic compartments in dendritic cells can result in HIV release upon interaction with T cells [110, 111]. However, these endocytic compartments were also described to be connected with the extracellular space [112, 113] and suggested to be invaginated domains distinct from classical endocytic vesicles [114]. Moreover, microvesicle release from T cells treated with ceramide inhibitors was not affected by such treatment [111], as previously reported for HIV-1 [115]. However, both viruses and microvesicles produced from ceramide-deficient cells failed to be captured by mature dendritic cells [111]. Therefore, more research is warranted on the specific sites of HIV assembly in particular cell types, and to what extent the endosomal compartments play a role in the HIV life cycle, as well as the possible convergence of HIV and shed microvesicle pathways.

It seems likely that HIV has simply adapted to use certain host factors for different exit modalities, and that these may vary among different types of cells, as well as under different conditions. It will be of continuing interest to further study the retroviral family, including the endogenous retroviruses, in order to determine whether the microvesicle cargo systems are perhaps a remnant of previous retroviral infections that happened earlier in evolution—and elements of which are now used in an opportunistic setting by retroviruses, such as HIV [56, 89–91, 102]. This overlap in pathways and the consequence of using overlapping machinery for release can result in phenotypic similarities between microvesicles and retroviruses and potentially interfere with anti-HIV strategies. For instance, HIV released from T cells has similar glycome properties as T-cell microvesicles, arguing for a common origin and indicating phenotypic similarity [116]. More research in the convergence of microvesicle and HIV pathways may improve our understanding of these processes and propel the development of new antiviral drugs directed against HIV.

The role of microvesicles during HIV infection has not yet been extensively studied, but they appear to be involved in both HIV infectivity enhancement and resistance depending on the cells of origin. Microvesicles derived from HIV-infected cells have been reported to contain HIV CCR5 coreceptors, allowing for enhanced HIV infection of other cells [34]. Moreover, microvesicles from megakaryocytes and platelets contain CXCR4 and upon transference confer susceptibility to cells normally resistant to HIV infection [35, 117]. In addition, during HIV replication the HIV Nef protein can alter the exosomal pathway by increasing the number of intracellular vesicles and MVBs [118–121]. HIV Nef-induced microvesicle release from infected and noninfected cells [39, 40] can induce apoptosis in CD4+ T cells [41] and convey resistance to HIV infection [61]. The transfer of Nef or other viral components through microvesicles may represent an important mechanism for immune evasion by viruses. In addition, exosomes can contain APOBEC3G, a cytidine deaminase that is part of the cellular antiviral system against retroviruses, which upon transference to recipient cells via exosomes can inhibit HIV replication [61]. While CD45, CD86, and MHC Class II molecules have been found in microvesicles from HIV-infected cells [42], possibly serving to silence the immune response, microvesicles derived from CD8+ T cells can act to suppress HIV replication [33]. Moreover, exosomes in association with HIV derived from dendritic cells significantly enhance HIV infection of CD4+ T cells [36]. In conclusion, microvesicles from HIV-infected cells as well as from noninfected cells play an important role in HIV replication and dissemination. Therefore, interference with microvesicle-mediated signaling could possibly be harnessed to halt HIV infection.

## 5. Retrotrasposon Elements and Microvesicles

Retrotransposon elements such as LINE, Alu, and human endogenous retroviruses (HERVs) make up about 45% of the human genome and have played an important role in genome evolution [122]. These viral-like elements infected germ cells in the human genome millions of years ago and then became a stable part of the inherited genetic material. Although most LINE elements are inactive, a number of active ones remain and are able to “jump” to new locations in the genome, contributing to genomic instability [123]. These events can have important effects on our genome, for example, by inactivating genes, altering gene expression and facilitating random insertion of new cDNA copies in the genome, as in integration of pseudogenes [124]. Many tumor cells also release retroviral-like microvesicles that contain active retrotransposon sequences, such as HERV-K [125].

Recently, tumor-derived microvesicles have been shown to be enriched in retrotransposon elements such as LINE1, Alu, and HERV-K [17]. Furthermore, HERV-K was transferred through microvesicles to normal HUVECs, which then showed an increase in HERV-K levels 12 hours following exposure to tumor microvesicles. In addition, the mouse retroviral RNA VL30 is packaged in retrovirus vectors by mouse packaging cell lines and transferred to human cells infected with those vectors [126]. The mouse VL30 has several stop codons in the regions encoding for genes such as *gag*, *pol* and *env*, thereby inhibiting its ability to encode functional proteins [126]. However, transfer of the VL30 mRNA together with tissue factor (TF) to human melanoma cells served to induce their metastatic potential. This change in phenotype apparently occurs through formation of a complex with the protein-associated splicing factor (PSF) protein which represses transcription of an insulin-like growth factor-1 (IGF-1) inducible gene, with dissociation of this complex allowing transcription to proceed [126]. Three of the 11 human genes affected by VL30 mRNA were oncogenic, suggesting that the transfer of retroviral RNA sequences can have catastrophic effects on recipient cells. Song et al. [126] have identified human retrotransposon sequences that are >90% identical to the mouse VL-30 suggesting human VL-30 transferred through microvesicles could have similar effects on transcription [126].

Long interspersed elements (LINEs)—most notably L1—comprise about 17% of the human genome. Several studies indicate that a subset of L1 elements is still actively expanding in the number of sequences within the human genome by retrotransposition. This active subpopulation, termed transcriptionally active (Ta), is approximately 2 million years old, and it has high levels of insertional polymorphism in the human population [127, 128]. Some of these new insertions may be intolerable and lethal and therefore eliminated; others may result in phenotypically tolerable disease, such as in Coffin-Lowry Syndrome and choroideremia [129–131], while still others have been associated with the induction of cancer, for example, lung cancer [132]. The high level of polymorphism of L1 elements indicates that they continue to have profound effects on the human genome, and recent evidence suggests that microvesicles may be a potential route of delivery for these elements [17]. This microvesicle-mediated Trojan Horse-like [92] transferance of transposons could perhaps allow for a stealthy dissemination of retrotransposons, especially in a tumor setting, avoiding immune-recognition, and achieving “long distance” delivery.

HERVs also entered the human genome millions of years ago and comprise about 8% of the human genome. They consist of *gag*, *pol,* and* env* sequences, flanked by two long terminal repeats [133]. Most of these sequences are now silent because of acquired mutations and deletions over the course of evolution, but HERV-K113 can produce intact, albeit noninfectious, retroviral particles [134]. Some of these sequences are still transcriptionally active and are associated with diseases, such as lymphoma and breast cancer [21, 135]. In cancer, hypomethylation of the genome seems to predominantly affect retrotransposon sequences (perhaps because they are highly abundant in the human genome), allowing increased transcription, especially in the case of the most recent entrants, which also happen to be the elements with the most intact coding potential [136]. Indeed retroviral-like microvesicles have been found in cancer patients, notably those with lymphomas [21], breast cancer [137], and teratomas [138]. As expected, these patients also had high levels of reverse transcriptase, and viral gag and env proteins and RNA in the tumor cells and retrovirus-like microvesicles released from them into the circulation [21]. Tumor microvesicles from cultured tumor cells also have been shown to be enriched in retrotransposon RNA, DNA, and reverse transcriptase, suggesting that a subpopulation of these microvesicles may indeed be of retroviral origin [19].

## 6. Concluding Remarks

In summary, this review deals with how extracellular vesicles—such as exosomes and shed microvesicles—share pathways with the assembly and release of retrotransposon elements and viruses. In Figure 1 we summarize how herpesviruses such as EBV and HSV, originate from the nucleus and can merge with microvesicle pathways. Several proteins used for exosome production are used by herpesviruses for functional release. Also, the convergence of these pathways may explain the observations of virus-like particles, which can be exosomes or shed microvesicles containing viral proteins or nucleic acids. Similar observations have been made for retroviruses and retrotransposon elements with circulating microvesicles containing retrotransposon RNA found in some cancer patients. It remains to be investigated to what extent exosomes and shed microvesicles are remnants of previous retroviral colonization. In this review we note the observations of retroviral as well as retrotransposon elements in microvesicles, perhaps allowing further dissemination of such nucleic acid sequences. The use of microvesicle pathway elements by viruses such as HIV may be suggestive of an intricate coevolution of different endogenous and exogenous (retro)virus subtypes. Viruses not only use microvesicle pathways for their own assembly and release but are also capable of exploiting the highly complex microvesicle communication system in an intercellular setting as simplified in Figure 2. During viral infection microvesicles can have various effects on different types of cells, either limiting viral infection or enhancing it. Thus, a picture is emerging that viruses and microvesicles are codependent pleiotropic entities. More research is needed into the differential functions of different subtypes of microvesicles and their cross-talk in relation to the immune response and outcome of viral infection.

## Figures and Tables

**Table 1:** Selective overview of viruses and vesicle function.

Virus	Immune status	Vesicle origin	Reference
HIV	Activating	CD8+ T cell	[33]
HIV	Activating	Megakaryocyte	[34, 35]
HIV	Activating	Dendritic cell	[36]
HIV	Evasion	Infected cell	[37–42]
CMV	Evasion	Infected cell	[43]
EBV	Evasion	Infected cell	[44–47]
HSV	Evasion	Infected cell	[37, 48–53]
